# IL-27 Mediates Pro-Inflammatory Effects *via* the ERK Signaling Pathway During Preterm Labor

**DOI:** 10.3389/fimmu.2021.709229

**Published:** 2021-10-08

**Authors:** Dongni Huang, Yuxin Ran, Zheng Liu, Jie He, Nanlin Yin, Hongbo Qi

**Affiliations:** ^1^ Department of Obstetrics, The First Affiliated Hospital of Chongqing Medical University, Chongqing, China; ^2^ Chongqing Key Laboratory of Maternal and Fetal Medicine, Chongqing Medical University, Chongqing, China; ^3^ Joint International Research Laboratory of Reproduction and Development of Chinese Ministry of Education, Chongqing Medical University, Chongqing, China; ^4^ Center for Reproductive Medicine, The First Affiliated Hospital of Chongqing Medical University, Chongqing, China

**Keywords:** IL-27/WSX-1, preterm labor, inflammation, ERK signaling pathway, mice model

## Abstract

Preterm labor (PTL) is a multifactorial syndrome that results in birth prior to 37 weeks of gestation. However, the specific molecular mechanisms underlying this condition have yet to be elucidated. Previous research demonstrated that the abnormal expression of IL-27, and its receptors, played a role in the pathophysiology of preterm labor. In the present study, we established a Lipopolysaccharide (LPS)-stimulated, infection-induced, preterm mouse model based on wild-type C57BL/6 mice and WSX-1^-/-^C57BL/6 mice. WSX-1 knockdown led to a significant delay in birth by 11.32 ± 2.157h. In addition, compared with wild-type C57B/6 mice, the expression levels of *IFN-γ, IL-1β, IL-6, TNF-α, and CXCL10*, in the fetal membrane and myometrium of WSX-1^-/-^mice were significantly lower, particularly in the myometrium. We also confirmed similar pro-inflammatory effects arising from IL-27 in human amniotic cell line (WISH) and human myometrial smooth muscle cell line (HMSMC). Once stimulated by LPS, the pro-inflammatory action exhibited a synergistic effect and appeared to be time-dependent. Finally, we demonstrated that LY3214996, an inhibitor of the ERK pathway, significantly inhibited the pro-inflammatory effect mediated by IL-27. Overall, our data confirmed that the inflammatory effect mediated by the IL-27/IFN-r/ERK axis is involved in preterm labor. Our findings, therefore, provide an enhancement in our etiological understanding of the mechanisms underlying PTL.

## Introduction

Preterm labor is defined as delivery within 37 weeks of gestation (1). According to global estimates by the World Health Organization (WHO), approximately 15 million neonates are born prematurely each year, thus accounting for 11.1% of all births (2). preterm labor is the only direct cause of 35% of neonatal deaths (3). In addition, preterm labor is associated with a range of long-term complications, including respiratory distress syndrome (4), neurological dysfunction (5), hearing loss, cerebral palsy (6), and visual impairment; collectively, these conditions bring heavy burden to both family and society (5). At present, the etiology of preterm labor is still unclear. In 2014, Romero et al. were the first to propose the concept of preterm labor as a multifactorial syndrome (5). Moreover, it is becoming clear that one of the main mechanisms associated with PTL is inflammation mediated by intrauterine infection (7).

Approximately 25% of premature babies are born with an amniotic infection (8); the ascending route is the most common route of infection. A previous study reported that the LPS-induced activation of uterine inflammation was sufficient to override the repressive effects of progesterone and induce a laboring phenotype (9). Pathogens and products are recognized by pattern recognition receptors (PRRs); these trigger the activation of the immune system and play a pro-inflammatory role by releasing cytokines (IL-1β, TNF-α, and IL-6), chemokines (CXCL11, CXCL10, and CXCL8), and prostaglandins. The activation of these specific signal transduction pathways causes uterine contractions, cervical structural changes, and weakened fetal membranes; ultimately, these events eventually lead to preterm labor (10).

The fetal membranes and myometrium are the main components of the maternal-fetal interface. These structures not only participate in signal transmission between the mother and fetus, but they are also important components in the maternal-fetal microenvironment (11). Previous studies have shown that amniotic epithelial cells are the first to be exposed to fetal signals (12). These amniotic epithelial cells are susceptible to hormones, mechanical tension, and other external stimuli; such susceptibility can result in the infiltration of a large number of immune cells and inflammatory mediators and can ultimately lead to preterm labor (13). Research has shown that LPS activates Toll-like receptors (TLR) through the NF-κB pathway in amniotic cells, thus releasing a range of secondary pro-inflammatory factors, including IL-6, IL-8, and MMP-9 in PTL (14). Similarly, when myometrial cells are stimulated by LPS, they can also release contraction-related proteins (CAP) (e.g., COX-2, CX-43, and OTR) and inflammatory mediators (e.g., IL-1β and TNF-α) (15). Therefore, epithelial cells in the amniotic membrane and myometrial cells may play an important role in the process of inflammatory imbalance in preterm labor.

Since the imbalance of inflammation is so important in the pathogenesis of preterm labor, it is vital that we develop an understanding of the specific molecular mechanisms involved. It is worth noting that pathogenic infection has been associated with increased levels of various pro-inflammatory cytokines, particularly IL-6 (16). Studies have also confirmed that the concentrations of IL-6 in cord blood from cases of preterm premature rupture of membranes (PPROM) are significantly increased; these high levels of IL-6 mediate a strong inflammatory response, stimulate the secretion of prostaglandin E2 (PGE2) and matrix metalloproteinases (MMPs), and play a key role in fetal membrane damage and rupture (17, 18). In addition, it is known that the level of IL-6 in fetal plasma is related to vaginitis and chorioamnionitis. Moreover, when IL-6 levels are higher than 11 pg/ml, it is also helpful to diagnose Fetal Inflammatory Response Syndrome (FIRS) (19). Therefore, IL-6 is an important biomarker for intrauterine infection-mediated inflammation imbalance. Based on present understanding, we hypothesized that IL-27, a member of the IL-6 family, will also play a role in preterm labor. In a previous study, Devergne et al. reported the expression levels of both IL-27 and IL-27R in extravillous trophoblast cells for the first time (20). Subsequent studies also reported that both IL-27 and WSX-1 are expressed in syncytiotrophoblast cells, extravillous trophoblast cells, and decidual cells (21–23). In addition, we reported that IL-27 can mediate inflammatory imbalances associated with Preeclampsia and preterm labor (23, 24). We were specifically interested in the fact that there was a significant increase of IL-27 levels in the peripheral blood of women with PROM. Therefore, we hypothesized that IL-27 also plays an important role in the pathophysiology of PTL.

In the present study, we used a variety of molecular biology techniques to investigate the specific mechanisms of how IL-27 might play a role in preterm labor, both *in vivo* and *in vitro*. Our results showed that IL-27 can promote the secretion of certain cytokines, particularly IFN-r in WISH and HMSMC cells, *via* the ERK signaling pathway, thus leading to an imbalance of inflammation that was associated with PTL. Our findings enrich our understanding of the etiology of preterm labor and provide new concepts for the diagnosis and treatment of this condition.

## Materials and Methods

### Mice

IL-27 receptor alpha chain-deficient mice on a C57BL/6 background (WSX-1^-/-^ mice) were purchased from The Jackson Laboratory in the USA. Wild-type C57BL/6 (WSX-1^+/+^ mice) were purchased from the Experimental Animal Center of Chongqing Medical University. The absence of the *WSX-1* gene was confirmed by western blotting and qRT-PCR. The WSX-1^-/-^ colony was housed together with a colony of wide-type C57BL/6 mice under specific pathogen-free conditions under a specific circadian cycle (12 h light/12 h dark). Animals had access to clean food and water *ad libitum.* Females (8-12 weeks of age) and male mice of the same genotype were mated in a chamber at a ratio of 2:1. Mating was confirmed by the presence of a vaginal plug at 8.00 am; the day of plug formation was counted as day 0.5 of pregnancy (dpc). Males were left with the females for a maximum of 2 days to ensure that the gestation date could be determined accurately. A rapid weight gain of more than 2 g from 12.5 dpc was deemed to confirm pregnancy. All operations were approved by the Animal Ethics Committee of Chongqing Medical University.

### Susceptibility to LPS

**Figure 1:**
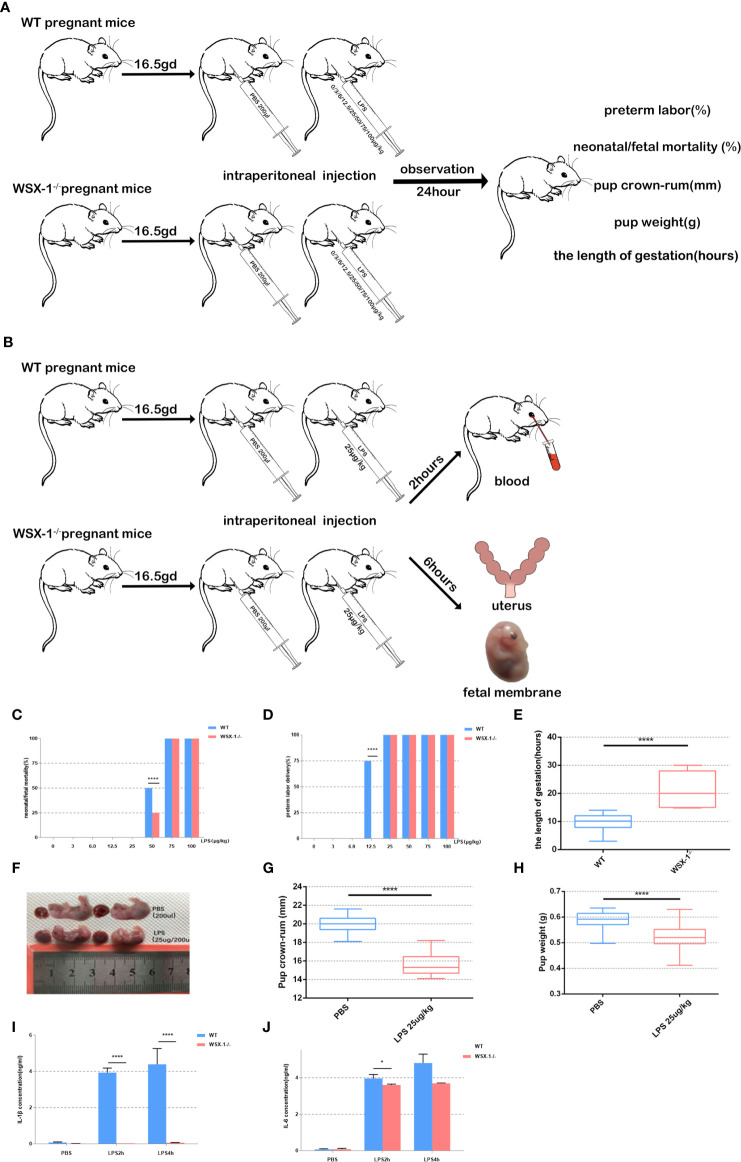
Flowchart showing the mouse model of preterm labor **(A, B)**. Reduced susceptibility to LPS in WSX-1^-/-^ mice. Statistical analyses of preterm labor rate and neonatal mortality in WSX-1^-/-^ and wild-type genotypes when treated with increasing concentrations of LPS for 24 h (0-100 µg/kg) (n = 5 mice for each concentration per group) **(C, D)**. 16.5gd mice were injected with 25 µg LPS/kg and monitored for delivery with video recordings **(E)**. Fetal body length and weight of preterm and control fetal mice **(E–H)**. IL-1β and IL-6 protein levels in peripheral blood from mice of both genotypes after treatment with different concentrations of LPS treatment (25 µg/kg) (n = 6), as determined by ELISA **(I, J)**. *P < 0.05, ****P < 0.0001.

On 16.5 dpc, mice were divided into 16 groups depending on the genotype and the concentration of LPS to be administered (n = 5 mice per group). LPS was administered to both wild-type and WSX-1^-/-^ mice *via* the intraperitoneal route using 200 μl of sterile phosphate buffer saline (PBS) containing 0 μg, 3 μg, 6 μg, 12.5 μg, 25μg, 50 μg, 75 μg, or 100 μg of LPS/kg. The various doses of LPS were randomly assigned to mice of the two different genotypes. We then recorded the timing of preterm delivery, neonatal/fetal mortality, birth weight, and pup crown-rump, for wild-type and WSX-1^-/-^ mice 24 h after receiving LPS (**Figure 1A**). Mice were monitored continuously and separately with closed-circuit television cameras and a digital video recorder.

### A Mouse Model of Preterm Labor Induced by LPS

On 16.5 dpc, mice were divided into four groups (n = 6 mice per group) and administered with different drug treatments: a group of wide-type mice were given 0 μg of LPS/kg (the WTN group), a group of wide-type mice were given 25 μg of LPS/kg (the WTP group), a group of WSX-1^-/-^ mice were given 0 μg of LPS/kg (the WSX-1^-/-^N group), and a group of WSX-1^-/-^ mice were given 25 μg of LPS/kg (the WSX-1^-/-^P group) (**Figure 1B**). Two hours after intraperitoneal injection, the mice were anesthetized with avertin, and blood was collected by eye puncture and placed into a 2 ml microcentrifuge tube. Serum was then separated from the maternal peripheral blood and stored at -20°C to await analysis. For tissue harvesting, a separate group of animals were euthanized 6 h after surgery (**Figure 1A**); most of the amnions and uterine tissues were recovered and immediately frozen in liquid nitrogen. Some of the amnions and uterine tissues were recovered and fixed in 4% paraformaldehyde for immunohistochemistry. We also ensured that all fetuses were counted and weighed.

### Cell Culture

The Human Amniotic Cell Lineage was obtained from the American Type Culture Collection (ATCC, UA). Amniotic cells were cultivated in Minimum Essential Medium(MEM) containing 10% FBS, 1% Non-essential Amino Acid Solution, 100 µg/ml of streptomycin and 100 U/ml of penicillin. Cells were maintained in a humid 5% CO2 atmosphere at 37°C and the medium was changed every 2 days. When the cells had grown to 80% confluency, the cells were treated with 1ml of 0.25% trypsin for 1 min and then 3 ml of MEM.

The Human Myometrial Smooth Muscle Cell Line was obtained from the American Type Culture Collection (ATCC, UA). HMSMCs were cultivated in Dulbecco’s Modified Eagle Medium (DMEM) containing 10% FBS, 100 µg/ml streptomycin, and 100 U/ml of penicillin. Cells were maintained in a humid 5% CO_2_ atmosphere at 37°C and the medium was changed every 2 days. When the cells had grown to 80% confluency, the cells were treated with 1 ml of 0.25% trypsin for 1 min and then 3 ml of DMEM.

### Reagents

Recombinant human IL-27 was purchased from R&D Systems (Minneapolis, MN, USA). Lipopolysaccharide was obtained from Sigma (USA). IL-6, IFN-γ, and IL-1β ELISA kits were purchased from Elabscience (USA). Beta-actin antibodies were purchased from Cell Signaling Technology (Danvers, MA, USA). A mouse IL-27 antibody was obtained from Abcam (Cambridge, MA, USA). The human WSX-1 antibody was obtained from Bioss (Beijing, China). p-ERK, T-ERK, p-JNK, and T-JNK antibodies were purchased from Santa Cruz Biotechnology (Dallas, TX, USA). The extracellular signal-regulated kinase (ERK) inhibitor (LY3214996) was purchased from MCE (USA). The concentration of DMSO was 0.1% (vol/vol) for all data subsets.

### Immunohistochemistry

Amniotic tissues were fixed in 4% paraformaldehyde, embedded in paraffin, and sectioned at a thickness of 5 μm. After deparaffinization in xylene and rehydration in a graded series of alcohol solutions, the sections were heated in sodium citrate buffer at 95°C for 20 min for antigen retrieval. The sections were then treated with 0.3% hydrogen peroxide in methanol for 20 min to block the activity of endogenous peroxidase. Then, 5% (wt/vol) BSA (Amresco, Solon, OH, UA)/PBS was used to block the slides for 1 h. Next, the samples were incubated at 4°C overnight with appropriate primary antibodies (1:50 dilution). The following morning, the sections were washed and incubated with a biotin-labeled secondary antibody (ZSGB-BIO, Beijing, China). The expression of IL-27 in amniotic tissues was detected by substrate chromatin 3- (3–)-diaminobenzidine (DAB) and hematoxylin (blue) staining. We also included negative controls that were not incubated with primary antibody. The stained sections were then evaluated by two observers using a Super Sensitive™ Link-Label IHC Detection System (BioGenex, San Ramon, CA, USA).

### Hematoxylin and Eosin (H&E) Staining

Paraffin sections were prepared as described above and stained with hematoxylin and eosin (H&E). After staining, we then used microscopy to evaluate the structure of the amniotic tissue.

### Immunofluorescence

WISH and HMSMC cells were grown in 24-well glass-bottomed dishes. Cells were treated with LPS (10ng/ml) or IL-27 (50 ng/ml) for 6 h or 15 min, fixed in 4% paraformaldehyde for 10 min, permeabilized with 0.1% Triton X-100 for 15 min, and blocked with 5% BSA for 1 h at 37°C. The cells were then incubated overnight at 4°C with primary antibodies at an appropriate dilution in blocking buffer. After overnight incubation, we performed three 10-min rinses with antibody wash solution (151 mM NaCl, 17 mM 263 trisodium citrate, and 0.05% TritonX-100). Cells were then treated with appropriate fluorescein-conjugated secondary antibodies against rabbit or mouse IgG. 6-diamidino-2-phenylindole (DAPI) was used for nuclear staining. We captured these Images under a fluorescence microscope(Life Technologies EVOS FL Auto, Beijing, China). The assay was repeated in triplicate.

### Western Blotting

Frozen tissue was removed from liquid nitrogen and immediately mixed with 500 μl of Radio Immunoprecipitation Assa (RIPA) lysis buffer containing Phenylmethylsulfonyl fluoride (PMSF) (at a proportion of 1:100). After homogenization with an IKA T10 basic ULTRA-TURRAX I (Germany), the tissue was centrifuged at 12000 rpm for 15 min to extract the supernatant. We then determined the concentration of total protein in each supernatant. Next, equal amounts of protein (20 μg) were separated by electrophoresis on 10% Sodium dodecyl sulfate (SDS) -polyacrylamide gels and blotted onto polyvinylidene fluoride (PVDF) membranes. Membranes were then blocked with 5% non-fat milk/PBS for 2 h and then incubated overnight with primary antibody. The next morning, the membranes were washed in PBS and incubated with an appropriate secondary antibody for 1 h. Finally, positive bands were detected using the ECL chemiluminescent detection system (GE Healthcare, Marlborough, MA, USA).

### Quantitative RT-PCR

Tissues and cells were lysed by Trizol in 2 ml microcentrifuge tubes in accordance with the manufacturer’s instructions. Next, we added 200 μl of chloroform, shook the samples vigorously for 15 s, incubated at room temperature for 2 min, and then centrifuged at 4°C at 12000 rpm for 10 min. The aqueous layer (containing the RNA) was extracted and mixed with 200 ml of isopropanol with gently shaking. After incubation for 10 min, the mixture was centrifuged at 4°C for 10 min to extract the precipitation which was then resuspended in DEPC water. Next, we determined RNA integrity by measuring the A260nm/A280nm ratio. Next, we reverse-transcribed the RNA with a PrimeScript RT Reagent Kit (Takara Bio Inc., Tokyo, Japan) in accordance with the manufacturer’s instructions. The primers used for qRT-PCR are listed in **Table 1** (Sangon Biotech, Shanghai, China). qRT-PCR was performed in an ABI PRISM 7000 system (Applied Biosystems, Foster City, CA, USA) with 2 µL of cDNA, 400 nm of each primer, 12.5 µL of Brilliant SYBR Green QPCR Master Mix (Takara Bio Inc., Tokyo, Japan), and 9.5 µL of ultra-pure water (a total volume of 25 µL per reaction) under the following conditions: 5 min at 95°C, 40 cycles of 30 s each at 95°C and 53°C, respectively; melting curves were generated after each endpoint amplification for 10 s at 95°C, followed by 30 s increments of 0.5°C from 65 to 95°C. *GAPDH* was used as an endogenous reference.

**Table 1:** Primers used for polymerase chain reaction (PCR).

Primers	Sequence
IL-1β F1	5′-CCTTAGGGTAGTGCTAAGAGGA-3′
IL-1β R1	5′-AAGTGAGTAGGAGAGGTGAGAG-3′
IFN-γ F1	5′-TCCTGTGACTGTCTCACTTAATC-3′
IFN-γ R1	5′-CTTAGGTTGGCTGCCTAGTT-3′
IL-6 F1	5′-CCTAGAGTACCTCCAGAACAGA-3′
IL-6 R1	5′-CAGGAACTGGATCAGGACTTT-3′
CXCL10 F1	5′-TCTCCCATCACTTCCCTACAT-3′
CXCL10 R1	5′-GGAGTAGTAGCAGCTGATTTGG-3′
MMP9 F1	5′-GGGCTTAGATCATTCCTCAGTG-3′
MMP9 R1	5′-GCCATTCACGTCGTCCTTAT-3′
MCP-1 F1	5′-GGCTGAGACTAACCCAGAAAC-3′
MCP-1 R1	5′-GAATGAAGGTGGCTGCTATGA-3′
TNF-α F1	5′-AGAGGGAGAGAAGCAACTACA-3′
TNF-α R1	5′-GGGTCAGTATGTGAGAGGAAGA-3′
GAPDH F1	5′-CAAGAGCACAAGAGGAAGAGAG-3′
GAPDH R1	5′-CTACATGGCAACTGTGAGGAG-3′

### ELISA

The serum levels of IL-6 and IL1β were detected with IL-6 and IL1β ELISA kits, respectively; these were purchased from R&D (Minnesota, USA). Optical absorbance was measured in a MultiskanGO plate reader (Thermo, Waltham, MA, USA) at 450 nm.

### Statistical Analysis

All data analysis was carried out with GraphPad Prism version 5.0 software (San Diego, CA, USA). Differences between groups were analyzed by randomized grouping analysis or by one-way analysis of variance (ANOVA). When ANOVA identified significant differences, we carried out a Bonferroni post-hoc test with Bonferroni correction to further test the differences among groups. P values < 0.05 were regarded as statistically significant.

## Results

### LPS-Induced Preterm Birth Mice With Intrauterine Inflammation

No preterm labors were recorded in the groups of mice receiving 0 μg, 3 μg, and 6 μg of LPS. At 12.5 μg of LPS/kg, the preterm labor rate of the wild-type mice was significantly higher than that of the WSX-1^-/-^ mice (P<0.0001). This suggests that WSX-1 deficiency may have potential effect on preterm labor. When stimulated with 50 μg of LPS/kg, the neonatal mortality rate of the wild-type mice was significantly higher than that of the WSX-1^-/-^ mice (**Figure 1C**, P<0.0001). As the dose of LPS increased, the preterm labor rate for both groups reached 100% (**Figure 1D**). In order to establish an effective and stable mouse model of preterm birth, on the basis of ensuring that both WSX-1-/- mice and WT mice will have preterm birth, and there will be differences in gestational weeks between the two groups. The most important thing is that neither mother nor fetus will die. Fortunately, at the concentration of 25 μg/kg, the duration of pregnancy in WSX-1^-/-^ mice after LPS injection was indeed prolonged 11.32 ± 2.157 hours (**Figure 1E**). Therefore, we chose to use 25 μg of LPS/kg as the optimal dose for subsequent experiments.

In addition, the body length of preterm fetal mice was lower than that in the PBS group (4.306 **±** 0.1767mm, P<0.0001) (**Figures 1F, G**). The same difference in weight of fetal mice**(** **Figure 1H**). We also found that the expression levels of IL-1β and IL-6 in the peripheral blood serum of WSX-1^-/-^ mice treated by LPS were significantly lower than those in the wild-type mice (**Figures 1I, J**).

### The Differential Expression of IL-27 in the Uterine and Fetal Membranes

**Figure 2:**
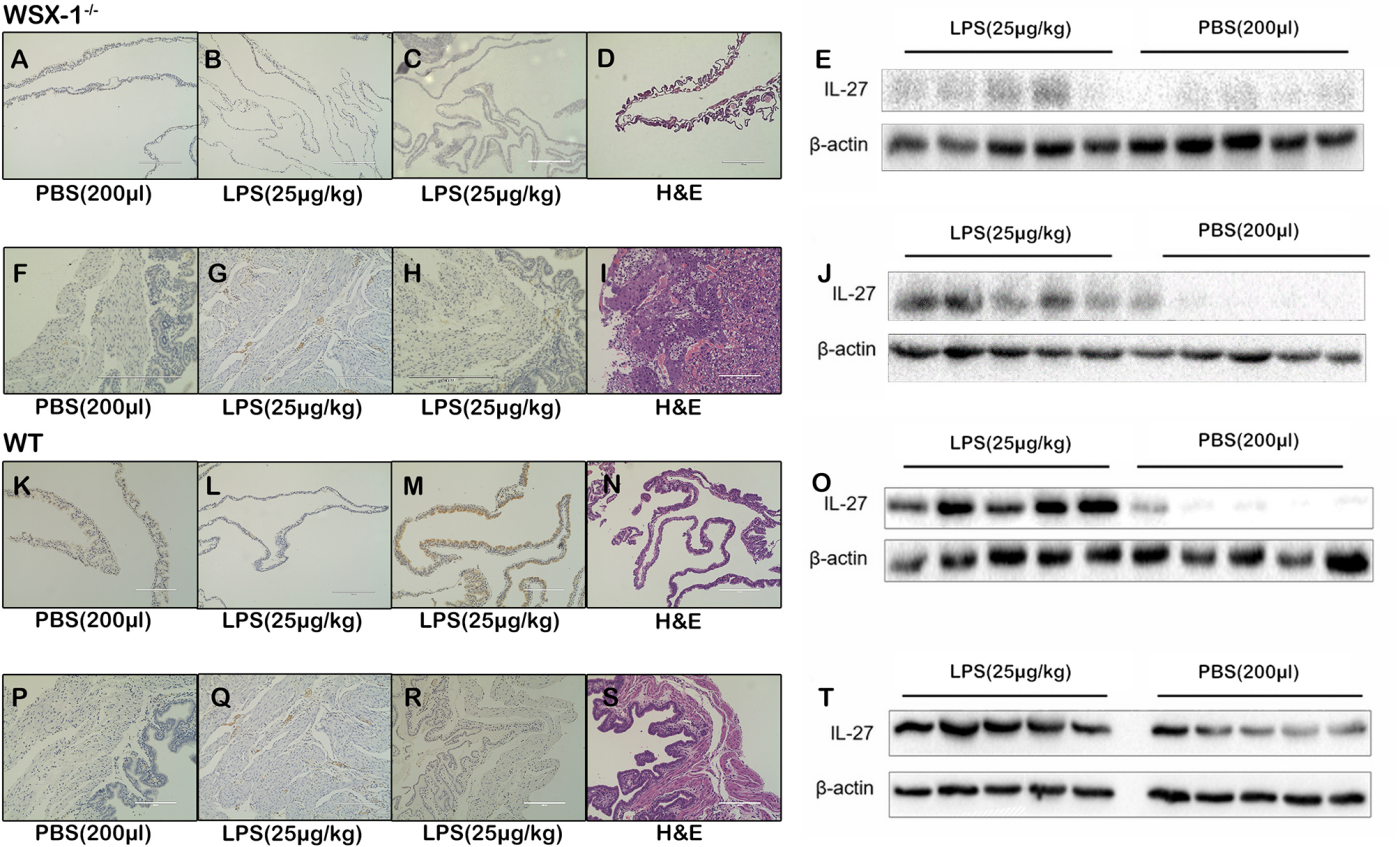
The role of IL-27 at the maternal-fetal interface in the WSX-1^-/-^ and wild-type mouse model of PTL. Immunohistochemical analysis of IL-27 protein expression and localization in **(A, C)** fetal-membranes and **(F, H)** uterus from WSX-1^-/-^ mice treated for 6 h with PBS or with LPS. Immunohistochemical analysis of IL-27 protein expression and localization in **(K, M)** fetal-membranes and **(P, R)** uterus from wild-type mice treated for 6 h with PBS or with LPS. H&E ensure the structure **(D, I, N, S)**. The others were controls for antibody specificity **(B, G, L, Q)**. Western blot analysis of IL-27 protein levels in **(E)** fetal-membranes and **(J)** uterus from WSX-1^-/-^ mice treated for 6 h with PBS or with LPS (25 µg/kg). Western blot analysis of IL-27 protein levels in **(O)** fetal-membranes and **(T)** uterus from WT mice treated for 6 h with PBS or with LPS (25 µg/kg). (n = 6 respectively).

Following the intraperitoneal administration of 25 μg of LPS/kg at 16.5 dpc, we observed that the expression of IL-27 at the fetal membrane of WSX-1^-/-^mice (**Figures 2A, C**). Moreover, the expression of IL-27 in fetal membranes of wild-type mice was significantly higher (**Figures 2K, M**). Western blot showed IL-27 was released under LPS induction (**Figures 2E**), and the concentration of IL-27 at the fetal membrane of wild-type mice was significantly higher than that of WSX-1^-/-^mice (**Figures 2E, O**). Similarly, Immunohistochemistry identified the location of IL-27 in uterus (**Figures 2F, H**), The Significant differences in expression of IL-27 can be seen in the uterus of wild-type mice (**Figures 2P, R**). The expression levels of IL-27 in the uterus of WSX-1^-/-^ mice were significantly increased following the intrauterine administration of LPS (**Figure 2J**). Wild-type mice also showed the same trend and the expression of IL-27 was also higher than which in WSX-1-/- mice (**Figures 2T**). H & E staining showed their structure (**Figures 2D, I, N, S**). The others were controls for antibody specificity (**Figures 2B, G, L, Q**).

### Inflammation Imbalance Was Observed in LPS-Induced WSX-1^-/-^ Mice

**Figure 3:**
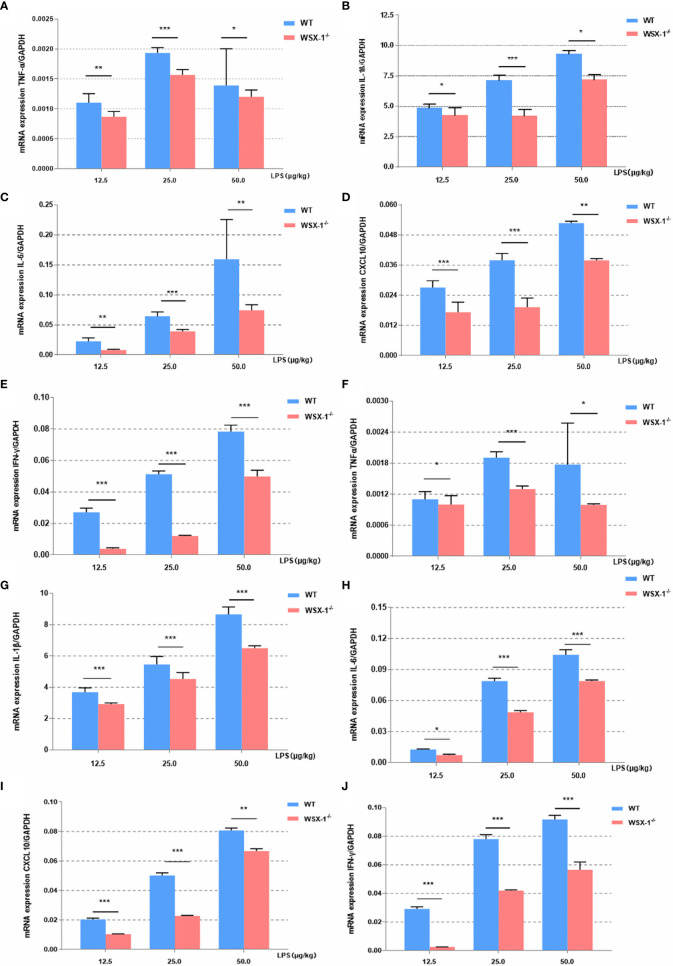
Inflammatory effects induced by LPS at the maternal-fetal interface from both genotypes. Concentration-dependent effects of LPS (12.5 µg/kg, 25 µg/kg, and 50 µg/kg) for 6 h on the mRNA expression of a range of key cytokines, including TNF-α, IL1β, IL6, IFN-γ, and CXCL10, in the fetal membranes **(A–E)** and uteri **(F–J)** of WT or WSX^-/-^ mice. Data are expressed as the mean ± SEM and the Student’s t-test. *P < 0.05, **P < 0.01 and ***P < 0.001 when compared between groups denoted by horizontal lines (n = 6).

Next, we compared differences in the inflammatory effect induced by LPS between the WSX-1^-/-^ mice and the wild-type mice. The expression levels of various inflammatory mediators were determined by qRT-PCR in uterine and fetal membrane tissues. Whether 12.5μg, 25μg or 50μg LPS/kg can lead to the release of various inflammatory mediators in membranes (**Figures 3A–E**). or uterus (**Figures 3F–J**). Moreover, the release of other inflammatory factors was positively correlated with the concentration of LPS except *TNF-α*. Analysis showed that the mRNA levels of *IL-6*, *IFN-γ*, *TNF-α*, *CXCL10*, and *IL-1β* in WT^-/-^ mice were significantly lower than those of wild-type mice after 12.5μg, 25μg and 50μg LPS/kg. This difference is even more pronounced at 25μg LPS/kg, which also confirms our previous dose selection.

### IL-27 Mediated Inflammation Imbalance in a LPS-Treated Human Amniotic Cell Line (WISH) and a Human Myometrial Smooth Muscle Cell Line (HMSMC)

**Figure 4:**
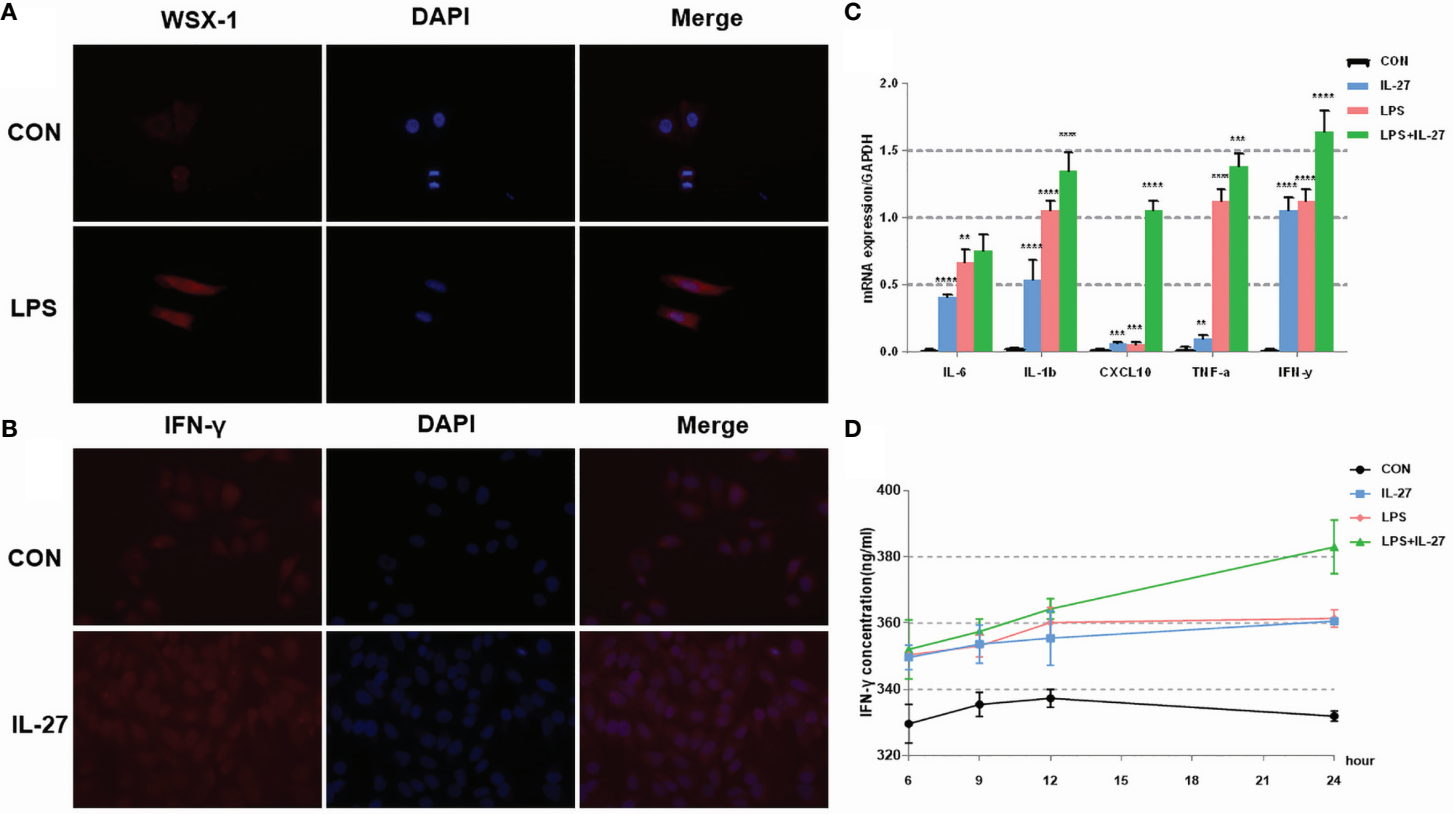
The expression and localization of WSX-1 and the inflammatory effects induced by IL-27 in WISH cells. **(A)** immunofluorescence analysis of WSX-1 protein expression and localization in WISH cells treated for 6 h with PBS or with LPS (1 µg/ml). **(B)** relative expression levels of several key cytokines, including TNF-α, IL1β, IL6, IFN-γ, and CXCL10, from WISH cells treated for 6 h without (CON) or with LPS (1 µg/ml) or IL-27 (50 ng/ml) or both, as measured by qRT-PCR. **(C)** Immunofluorescence analysis of IFN-γ protein expression and localization in WISH cells after IL-27 (50 ng/ml) treatment for 6 h **(D)** time-dependent effects of 50 ng/mL of IL-27, 1 µg/ml of LPS, or both of these factors, of those on the expression of IFN-γ, as measured by ELISA. **P < 0.01, ***P < 0.001, ****P < 0.0001.

**Figure 5:**
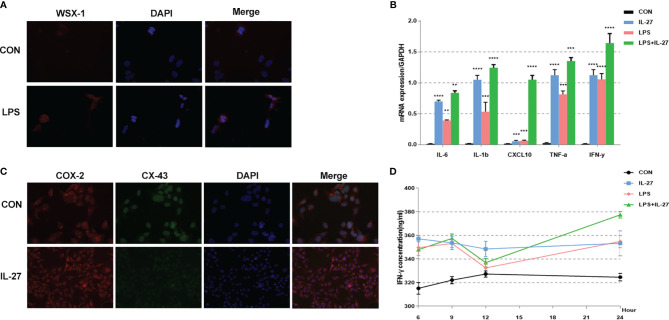
Expression and localization of WSX-1 and iInflammatory effects induced by IL-27 in HMSMC cells. **(A)** Immunofluorescence analysis of WSX-1 protein expression and localization in HMSMC cells treated for 6 h without (PBS) or with LPS (1 µg/ml). **(B)** Relative expression of a range of key cytokines, including TNF-α, IL1β, IL6, IFN-γ, and CXCL10, in WISH cells treated for 6 h without (CON) or with LPS (1 µg/ml) or IL-27 (50 ng/ml) or both factors, as measured by qRT-PCR. **(C)** double labeling and immunofluorescence analysis of CX-43 and COX-2 protein expression and localization in cells treated with or without IL27 (50 ng/ml) treatment for 6 h Bar = 50 μm. **(D)** Time-dependent effects of 50 ng/mL IL-27, 1 µg/ml LPS, or both factors, on the expression of IFN-γ, as determined by ELISA. **P < 0.01, ***P < 0.001, ****P < 0.0001.

To further confirm the observed IL-27-mediated inflammation imbalance, we next performed *in vitro* experiments using WISH and HMSMC cell lines. First, we investigated changes in the expression of WSX-1 in both cell lines following the administration of LPS. After 6 h of treatment with LPS (1 μg/mL), the protein expression levels of WSX-1 increased significantly (**Figures 4A**, **5A**). To determine the extent of IL-27-induced inflammation, we stimulated these cells with LPS (1 μg/mL), IL-27 (50 ng/mL), IL-27 (50 ng/mL) + LPS (1 μg/mL) and used quantitative RT-PCR to determine the levels of various downstream mediators of inflammation. Interestingly, the levels of IFN-γ, TNF-α, IL-1β, and CXCL10, were all augmented. Moreover, the synergistic effect of IL-27 and LPS amplified the inflammatory cascade induced by LPS at the maternal-fetal interface by 1.45-fold, 1.13-fold, 1.28-fold, and 9.16-fold, respectively. A similar inflammatory response was also observed in HMSMC cells (**Figures 4B**, **5B**). Of the increased inflammatory mediators, IFN-γ showed the largest increase in both cell lines and was very stable. The expression levels of IFN-γ in the supernatant of WISH and HMSMC cells were tested by ELISA following treatment with LPS alone, IL-27 alone, and LPS + IL-27, in a time-dependent manner. The treatment groups all showed different patterns of IFN-γ increase; however, the IL-27 + LPS group showed the greatest increase in IFN-γ expression after 24 h of treatment (**Figures 4D**, **5D**). These results were further confirmed by immunofluorescence (**Figure 4C**). In addition, the treatment of HMSMC cells with IL-27 led to an increase in the expression levels of classic contractile proteins (e.g., COX-2 and CX-43), thus implying that IL-27 may be involved in preterm delivery by mediating uterine contractions (**Figure 5C**).

### IL-27 Mediated Inflammation Imbalance *via* the ERK Signaling Pathway

**Figure 6:**
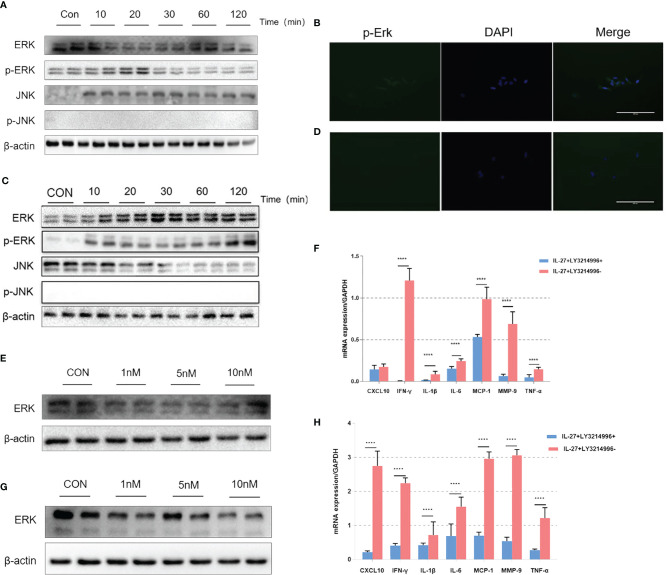
The effects of IL-27 on pro-inflammatory effects *via* a dominant ERK pathway in WISH cells and HMSMC cells. Analysis of WISH cells with regards to activated or tyrosine phosphorylated ERK (p-ERK) and JNK (p-JNK) proteins by western blotting **(A)** and immunofluorescence **(B)**. The expression of ERK with 0 nM, 5 nM, and 10 nM LY3214996, as determined by western blotting **(E)**. The relative expression levels of IFN-γ after 24 h incubation with 10nM LY3214996, as determined by qRT-PCR **(F)**. The same analysis has repeated in HMSMC cells **(C, D, G, H)**. ****P < 0.0001.

Previous data showed that IL-27 could release various inflammatory mediators such as IL-6, IFN-γ, TNF-α, IL-1β, MCP-1, MMP9 and CXCL10 mainly *via* the ERK and JNK signaling pathways (24, 25). Therefore, we investigated the inflammatory imbalance mediated by IL-27 in WISH and HMSMC cells. Cells were treated with IL-27 (50 ng/ml) at specific time points used western blotting to determine the levels of phosphorylated ERK (p-ERK) and JNK (p-JNK) proteins. Our analysis showed that IL-27 significantly induced the phosphorylation of ERK in WISH and HMSMC cells in a time-dependent manner (**Figures 6A, C**) but not the JNK pathway. Immunofluorescence microscopy further confirmed the activation of the ERK signaling pathway (**Figures 6B, D**). Moreover, the administration of LY3214996, an inhibitor of the ERK pathway, led to a significant inhibition in the release of various inflammatory mediators induced by IL-27 (**Figures 6E, G**), especially with regards to IFN-γ (**Figures 6F, H**).

## Discussion

Preterm labor is a process of inflammatory imbalance that involves inflammatory mediators (26, 27). Our previous data indicated that the abnormal expression levels of IL-27 in human tissues were related to the pathogenesis of preterm labor, particularly in the fetal membrane. However, this has not been verified *in vivo*, and the specific molecular mechanisms underlying these observations have yet to be elucidated. In the present study, we identified several new findings: 1) The rates of preterm labor and neonatal mortality in the wild-type mice were higher than that of the WSX-1-deficient mice when treated with LPS; 2) The mRNA levels of IL-6, IFN-γ, TNF-α, IL-1β, and CXCL10, in the fetal membranes and myometrium of the WSX-1^-/-^ mice when treated with LPS were significantly lower than in the group of wild-type mice, especially for IFN-γ; 3) IL-27 synergistically enhanced the expression of downstream inflammatory mediators that were stimulated by LPS in WISH and HMSMC cell lines, thereby amplifying the pro-inflammatory effect; 4) By applying specific signaling pathway inhibitors, we found that IL-27-mediated inflammation occurred mainly *via* the ERK signaling pathway in WISH and HMSMC cell lines.

The LPS-induced mouse represents a classical model of preterm labor. At first, We tried to find the inflection point where WSX-1 deficiency affects preterm birth. According to the results, it is likely to be 12.5μg/kg. However, 12.5μg LPS/kg cannot induce 100% preterm labor in all groups. In the present study, 25 μg of LPS/kg was dissolved in 200 μl of PBS and administered as intraperitoneal injections into wild-type and WSX-1^-/-^ mice. The reasons for this choice are as follows: Firstly, there are no deaths of mice (both mothers and fetuses) at this dose. Secondly, using this dose both groups will induce preterm labor. Lastly, Compared with the wild-type mice, WSX-1^-/-^ took the mice more 11.32 ± 2.157 hours to begin delivery. Which reflected that IL-27 had a potential effect on pregnancy maintenance. In other words, our study not only focused on the inflection point of the incidence of preterm delivery, but also pays more attention to how to prolong the gestational age. Similarly, IL-6, a member of the same family as IL-27, has also been confirmed to be involved in preterm labor. Robertson et al. administered mice with intraperitoneal injections of 2.0 μg of LPS in 200 μl of PBS; these authors found that IL-6 null mutant mice were delivered 24 h later than wild-type mice (28). However, these findings should be interpreted cautiously; there are many differences between mice and humans with regards to anatomy.

Inflammatory imbalance plays a key role in the pathogenesis of preterm labor. In the present study, we investigated the downstream inflammatory mediators that were activated by IL-27 after LPS stimulation *in vivo*. The expression levels of *IL-6, IFN-γ, TNF-α, CXCL10,* and *IL-1β*, in WSX-^1-/-^ mice were all significantly lower than that those in wild-type mice following LPS stimulation. Similarly, IL-27 caused a similar inflammation imbalance in both WISH and HUMSC cells. Data derived from both our *in vivo* and *in vitro* experiments demonstrated a significant increase in IFN-γ, a pleiotropic cytokine that is involved in both acquired immunity and innate immune and associated with various autoinflammatory and autoimmune diseases (29). Elevations of IFN-γ in the peripheral blood have been reported in both Preeclampsia (PE) and Gestational Diabetes Mellitus (GDM) (30, 31). Studies have indicated that IFN-r may affect vascular recasting by regulating the levels of EPHB4 and IGF-1/2, thus leading to PE (32, 33). As an inflammatory disease, preterm labor is also related to IFN-γ. Significant increases in the levels of IFN-γ in the amniotic fluid of women at term were first reported in 1996 (34). Subsequently, a series of studies showed that the levels of IFN-γ in maternal plasma and cord blood were also related to preterm labor (35, 36). Schust et al. reported that IFN-γ produced by activated iNKT cells may be related to the activation of other immune cells, thus further amplifying the inflammatory effect that leads to PTL. However, contradictory reports are also evident in the literature. For example, some authors highlighted the fact that IFN-γ plays a key anti-inflammatory role during preterm labor (37, 38). In short, the specific molecular mechanisms underlying the action of IFN-γ in the pathophysiology of preterm labor remains unclear and further research is vital.

Previous studies showed that IL-27 regulates IFN-γ from both pro-inflammatory and anti-inflammatory aspects (39). The pro-inflammatory effect has been extensively studied. For example, when affected with *Toxoplasma gondii*, WSX-1^-/-^ mice produced a strong IFN-γ response that led to fatal inflammation (40). In contrast, when mediated by IL-27, IFN-γ can also play an anti-inflammatory effect. WSX-1^-/-^ mice are known to be more susceptible to Leishmania due to the inhibition of interferon-γ production (41, 42). It follows, therefore, that IFN-r may represent a double-edged sword. It appears that the microenvironment determines its specific inflammatory effect. We have previously reported that IL-27 regulates IFN-γ-mediated pro-inflammatory effects in PE and that the IL-27/IFN-γ axis also plays a pro-inflammatory effect in preterm labor (43); our present findings concur with these previous findings.

Since IL-27-mediated inflammation imbalance plays an important role in preterm labor, we wanted to identify the specific molecular mechanisms that are involved in this pathway. By using different pathway inhibitors, we found that IL-27 mainly activates the ERK signaling pathway in WISH and HMSMC cells, thus triggering an inflammatory cascade that ultimately leads to preterm labor. Moreover, we will try to find the expression of p-ERK in fetal membrane and uterus of WSX-1-/- mice was lower than WT mice in further study. The extracellular signal-regulated kinase (ERK) pathway is a member of the mitogen-activated protein kinase (MAPK) superfamily. The ERK signaling pathway plays an important role in mediating cell migration, division, and survival, and has been widely reported to mediate diseases of the cardiovascular, nervous, and immune systems, as well as tumors and other diseases (39, 44, 45). *In vivo* experiments involving rat and mice models, and *in vitro* experiments involving fetal membranes and myometrium, have confirmed that activation of the ERK signaling pathway is closely related to the pathogenesis of preterm labor, particularly with regards to inflammatory aspects (24, 46–48). Sepsis is associated with an increase in phosphorylated extracellular signal-regulated kinase (p-ERK) which can be used as a therapeutic target for sepsis (49). The inhibition of ERK can also reduce the production of IL-23 and IL-1β and reduce the symptoms of autoimmune diseases such as demyelinating diseases (50), colitis (51), and rheumatoid arthritis (52). In an earlier study, we showed that IL-27 could mediate an excessive inflammatory response in whole fetal membranes *via* the ERK signaling pathways, thus contributing to preterm labor (13). Moreover, Yao et al. previously reported that the IFN-γ-induced production of invariant natural killer T cells plays an important role in inflammation-induced preterm labor by activating the ERK pathways (53). LY3214996 is a highly selective inhibitor of ERK1 and ERK2 and exhibits similar or higher levels of anti-tumor activity and anti-inflammatory effects when compared with other ERK inhibitors (54). In the present study, LY3214996 significantly suppressed the inflammatory response, particularly the production of IFN-γ induced by IL-27. Accordingly, we speculate that IL-27 might cause cytokine storms by activating the ERK pathway to release a large number of proinflammatory factors under the regulatory control of IFN-γ. Our data suggest that LY3214996 might effectively reverse or alleviate the severity of such cytokine storms, thus enriching the development of theoretical strategies for the treatment of preterm labor.

It is worth noting that LPS has always been considered to play an important role in preterm birth caused by inflammatory imbalance. Compared with LPS, other inflammatory mediators play a relatively mild role which IL-27 is no exception. Therefore, we need to interpreted the results very carefully. The previous studies have reported that IL-27 has both pro-inflammatory and anti-inflammatory properties. When stimulated by external pathogens, such as LPS, IL-27 can exert a pro-inflammatory response, and by activating Th1 cells, can also mediate immune activation in diseases. In addition, IL-27 can also increase the content and ratio of Th1 and Th17 cells, thus leading to the excessive secretion of inflammatory mediators in local tissues, such as IFN-γ, CXCL10, and TNF-α, Thereby forming an inflammatory cascade that eventually induces autoimmune diseases (55, 56). However, when there are pro-inflammatory effects in the external environment, the anti-inflammatory effects of IL-27 can also be triggered (57, 58). Finally, this study has certain limitations. For example, considering the consistency of the experiment and the limitations of the condition, we applied two different cell lines instead of using primary cells, but we do believe that it can still bring some new information to a certain extent. In conclusion, we found that IL-27 mediates the inflammatory cascade by activating the ERK pathway in the fetal membrane and myometrium, thereby influencing the remodeling process in the fetal membrane tissue and the contraction of the myometrium, thus participating in the pathogenesis of preterm labor. This study may further enrich the inflammatory pathogenesis of preterm labor and facilitate the development of novel diagnostic and treatment strategies.

## Data Availability Statement

The raw data supporting the conclusions of this article will be made available by the authors, without undue reservation.

## Ethics Statement

The animal study was reviewed and approved by the Animal Ethics Committee of Chongqing Medical University.

## Author Contributions

DH, NY, and HQ conceived the study. DH and YR collected the data. DH, YR, ZL, JH, and NY performed the statistical analysis. All authors contributed to the article and approved the submitted version.

## Funding

This research was supported by a grant from the National Natural Science Foundation of China (References: 81801483).

## Conflict of Interest

The authors declare that the research was conducted in the absence of any commercial or financial relationships that could be construed as a potential conflict of interest.

## Publisher’s Note

All claims expressed in this article are solely those of the authors and do not necessarily represent those of their affiliated organizations, or those of the publisher, the editors and the reviewers. Any product that may be evaluated in this article, or claim that may be made by its manufacturer, is not guaranteed or endorsed by the publisher.
